# Characterization of CCoV-HuPn-2018 spike protein-mediated viral entry

**DOI:** 10.1128/jvi.00601-23

**Published:** 2023-09-28

**Authors:** Yongmei Liu, Danying Chen, Yuanyuan Wang, Xinglin Li, Yaruo Qiu, Mei Zheng, Yanjun Song, Guoli Li, Chuan Song, Tingting Liu, Yuanyuan Zhang, Ju-Tao Guo, Hanxin Lin, Xuesen Zhao

**Affiliations:** 1 Beijing Key Laboratory of Emerging Infectious Diseases, Institute of Infectious Diseases, Beijing Ditan Hospital, Capital Medical University, Beijing, China; 2 Beijing Institute of Infectious Diseases, Beijing, China; 3 National Center for Infectious Diseases, Beijing Ditan Hospital, Capital Medical University, Beijing, China; 4 National Key Laboratory of Intelligent Tracking and Forecasting for Infectious Diseases, Beijing, China; 5 Peking University Ditan Teaching Hospital, Beijing, China; 6 Baruch S. Blumberg Institute, Hepatitis B Foundation, Doylestown, Pennsylvania, USA; 7 Department of Medical Genetics, University of Alberta, Edmonton, Alberta, Canada; 8 Molecular Genetics Laboratory, Alberta Precision Laboratories, Edmonton, Alberta, Canada; Loyola University Chicago, Maywood, Illinois, USA

**Keywords:** CCoV-HuPn-2018, coronavirus, virus entry, APN, IFITM

## Abstract

Canine coronavirus-human pneumonia-2018 (CCoV-HuPn-2018) was recently isolated from a child with pneumonia. This novel human pathogen resulted from cross-species transmission of a canine coronavirus. It has been known that CCoV-HuPn-2018 uses aminopeptidase N (APN) from canines, felines, and porcines, but not humans, as functional receptors for cell entry. The molecular mechanism of cell entry in CCoV-HuPn-2018 remains poorly understood. In this study, we demonstrated that among the nine APN orthologs tested, the APN of the Mexican free-tailed bat could also efficiently support CCoV-HuPn-2018 spike (S) protein-mediated entry, raising the possibility that bats may also be an alternative host epidemiologically important for the transmission of this virus. The glycosylation at residue N747 of canine APN is critical for its receptor activity. The gain of glycosylation at the corresponding residues in human and rabbit APNs converted them to functional receptors for CCoV-HuPn-2018. Interestingly, the CCoV-HuPn-2018 spike protein pseudotyped virus infected multiple human cancer cell lines in a human APN-independent manner, whereas sialic acid appeared to facilitate the entry of the pseudotyped virus into human cancer cells. Moreover, while host cell surface proteases trypsin and TMPRSS2 did not promote the entry of CCoV-HuPn-2018, endosomal proteases cathepsin L and B are required for the entry of CCoV-HuPn-2018 in a pH-dependent manner. IFITMs and LY6E are host restriction factors for the CCoV-HuPn-2018 entry. Our results thus suggest that CCoV-HuPn-2018 has not yet evolved to be an efficient human pathogen. Collectively, this study helps us understand the cell tropism, receptor usage, cross-species transmission, natural reservoir, and pathogenesis of this potential human coronavirus.

**IMPORTANCE:**

Viral entry is driven by the interaction between the viral spike protein and its specific cellular receptor, which determines cell tropism and host range and is the major constraint to interspecies transmission of coronaviruses. Aminopeptidase N (APN; also called CD13) is a cellular receptor for HCoV-229E, the newly discovered canine coronavirus-human pneumonia-2018 (CCoV-HuPn-2018), and many other animal alphacoronaviruses. We examined the receptor activity of nine APN orthologs and found that CCoV-HuPn-2018 utilizes APN from a broad range of animal species, including bats but not humans, to enter host cells. To our surprise, we found that CCoV-HuPn-2018 spike protein pseudotyped viral particles successfully infected multiple human hepatoma-derived cell lines and a lung cancer cell line, which is independent of the expression of human APN. Our findings thus provide mechanistic insight into the natural hosts and interspecies transmission of CCoV-HuPn-2018-like coronaviruses.

## INTRODUCTION

Coronaviruses (CoVs) are a large group of single-stranded enveloped positive-sense RNA viruses with a broad host range in mammalian and avian species and have been identified as etiological agents of multiple emerging infectious diseases (1 – 3). CoVs can be divided into four genera: alphacoronavirus, betacoronavirus, gammacoronavirus, and deltacoronavirus (2). At least seven human CoVs (HCoVs) have been identified so far. Except for 229E and NL63, which are alphacoronaviruses, the other HCoVs, OC43, HKU1, severe acute respiratory syndrome coronavirus (SARS-CoV), Middle East respiratory syndrome coronavirus (MERS-CoV), and SARS-CoV-2, are all betacoronaviruses. 229E, NL63, OC43, and HKU1 have been circulating in humans for many years and mainly cause mild upper respiratory tract infections, which typically self-recover within 1 week (4). SARS-CoV, MERS-CoV, and SARS-CoV-2 just emerged in the human population in the past 20 years and were highly pathogenic (2, 4). For example, the ongoing pandemic SARS-CoV-2 was first reported in 2019 and will have caused ~761 million confirmed cases and ~6.9 million confirmed deaths in the world by 21 March 2023 (https://www.who.int/emergencies/diseases/novel-coronavirus-2019). It is generally believed that these newly emerging HCoVs originated from bat CoVs and jumped to humans *via* an intermediate host, e.g., a civet cat for SARS-CoV and a dromedary camel for MERS-CoV (5, 6).

In addition to these seven well-recognized HCoVs, occasional spillover of common animal coronaviruses to humans has been noticed (7). One example is porcine deltacoronavirus (PDCoV), which was recently isolated from three Haitian children with acute undifferentiated febrile illness (8). The other example is canine alphacoronavirus, which was recently isolated from pediatric and adult patients with fever, malaise, or pneumonia in Haiti and Malaysia (9, 10). Current evidence suggests that such spillover events have occurred multiple times in different countries over the past 20 years (7). These findings highlight the continuing threat to human health posed by the cross-species transmission of zoonotic coronaviruses and warrant further studies on the surveillance of animal CoVs in humans, their human-to-human transmissibility, and their pathogenesis.

Virus entry is a key determinant of host range, cell tropism, pathogenesis, and cross-species transmission (11 – 13). As a class I viral fusion protein, CoV spike (S) protein contains an extracellular S1 subunit that binds to cellular receptors and a membrane-anchored S2 subunit that is responsible for membrane fusion (14). Membrane fusion occurs either at the plasma membrane in a pH-independent manner or in the endocytic compartments in a pH-dependent manner (15, 16). The efficiency of virus entry is determined by many viral and host cellular factors, including receptor usage, binding to attachment factors, susceptibility of S proteins to protease cleavage, and/or acid-induced conformation changes (15, 17). While SARS-CoV, SARS-CoV-2, and HCoV-NL63 share angiotensin-converting enzyme 2 (ACE2) as the entry receptor (18 – 20), MERS-CoV employs dipeptidyl peptidase-4 (DPP4) as a receptor for host cell entry (21). Of note, several alpha coronaviruses, including HCoV-229E, feline coronavirus (FCoV), canine coronavirus (CCoV), and porcine CoVs [transmissible gastroenteritis virus (TGEV) and porcine epidemic diarrhea virus (PEDV)], use aminopeptidase N (APN) as receptors (22 – 26). Interestingly, some CoVs, such as HCoV-OC43, HCoV-HKU1, and bovine CoV, bind to 9-O-acetylated sialic acids *via* a conserved receptor-binding site in domain A within the S1 subunit of spike protein to initiate the infection (27). In addition, CoV entry requires S protein priming by multiple proteases, including furin, cell surface transmembrane proteases (TMPRSS), trypsin, and endosomal cathepsins, which cleave S protein at the S1/S2 boundary or S2′ cleavage site, to induce membrane fusion (17, 28).

**FIG 1:**
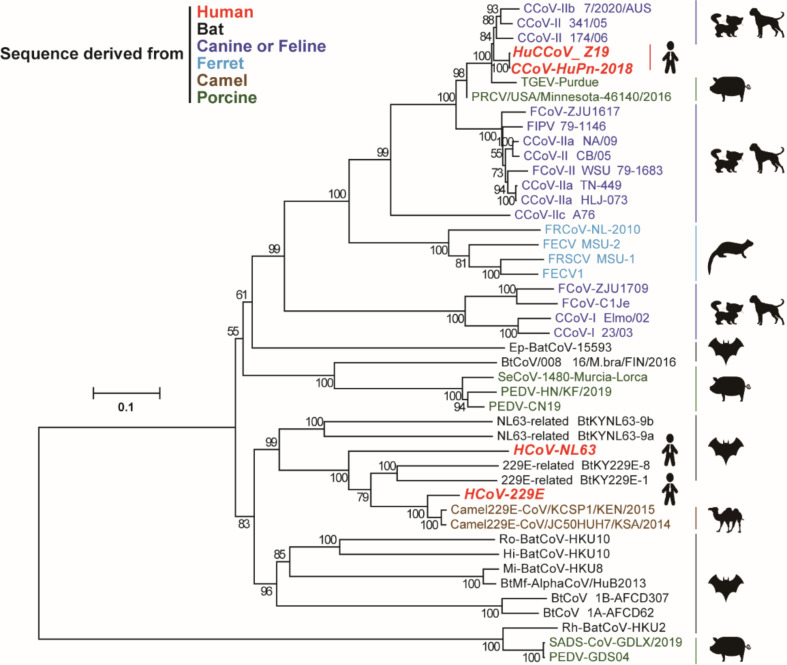
Evolutionary relationship between CCoV-HuPn-2018 and other alphacoronaviruses. The phylogenetic tree was constructed based on amino acid sequences of S proteins using the neighbor-joining method implemented in MEGA7.0.26 software. The scale bar indicates amino acid substitutions per site. The percentage of replicate trees in which the associated taxa clustered together in the bootstrap test (1,000 replicates) is shown next to the branches. The newly emerged CCoV-HuPn-2018 and Haitian strain HuCCoV_Z19 are highlighted in red like other HCoVs. BatCoV, bat coronavirus; PRCV, porcine respiratory coronavirus; SADS-CoV, swine acute diarrhea syndrome coronavirus; SeCoV, swine enteric coronavirus.

Canine coronavirus-human pneumonia-2018 (CCoV-HuPn-2018) is the human isolate of CCoV, which was recently isolated from a Malaysian child with pneumonia (9). This virus can be considered a potential human CoV. Sequence analysis suggests that CCoV-HuPn-2018 might be generated by multiple recombination events between CCoVs and feline CoVs (9). Since the S protein of CCoV-HuPn-2018 is phylogenetically close to the S proteins of CCoVs, FCoVs, and TGEVs but not the two other human alphacoronaviruses 229E and NL63 (Fig. 1), CCoV-HuPn-2018 uses the APN of a domestic dog, domestic cat, or farmed pig, but not a human, as receptors for cell entry *in vitro* (29). So far, the cell entry process of this potential human CoV remains poorly understood. Many questions remain unanswered, e.g., if human APN (hAPN) cannot be used as an entry receptor, how does CCoV-HuPn-2018 enter human cells and establish infection?

In this study, we used a vesicular stomatitis virus (VSV)-based pseudotyped virus system of CCoV-HuPn-2018 to examine the receptor activity of nine APN orthologs, cell tropism, entry route, host factors, and host restriction factors for cell entry. Our findings provided valuable insights into the cell entry and interspecies transmission of this novel potential human CoV.

## RESULTS

### Receptor activities of nine animal APNs

**FIG 2:**
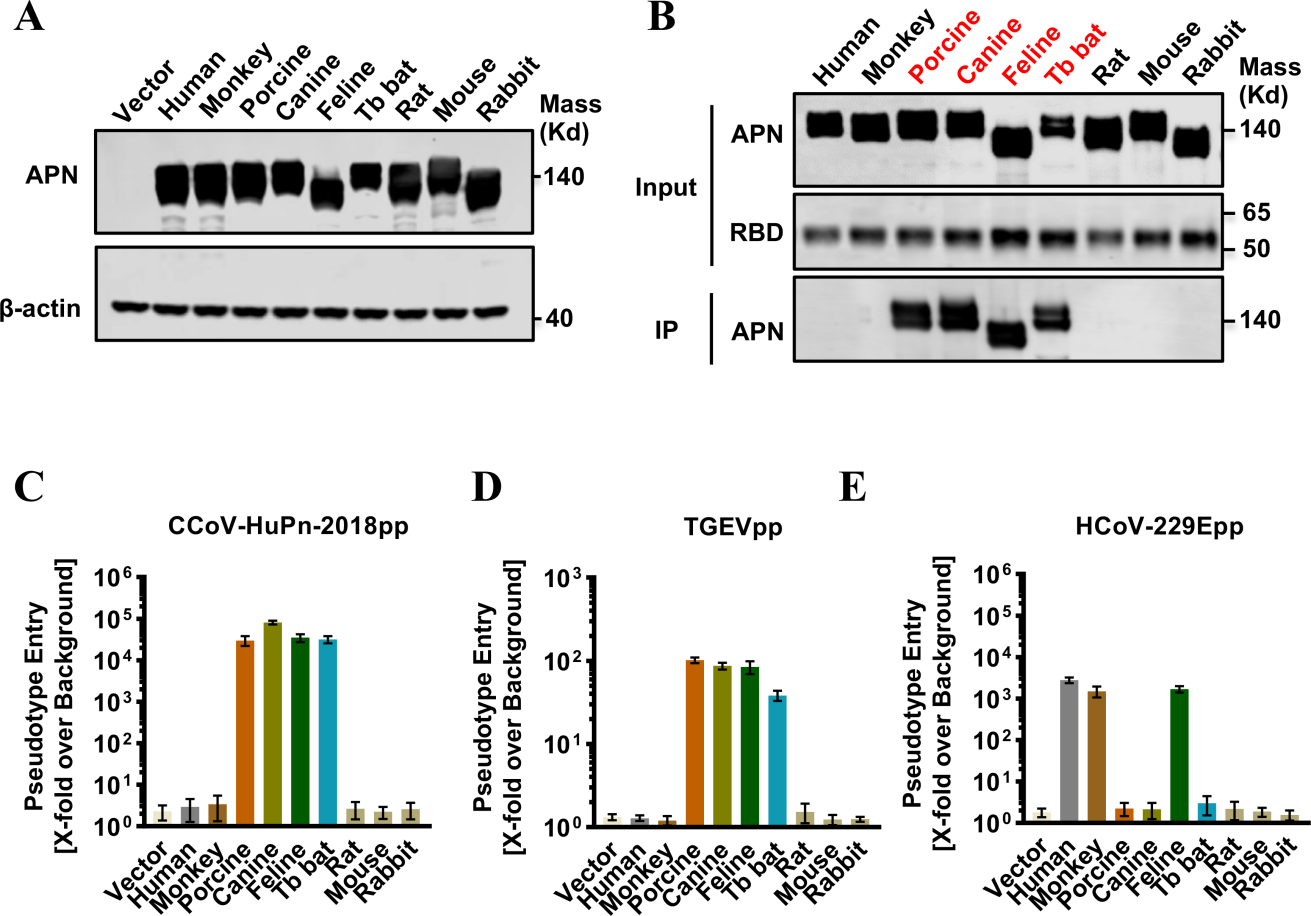
Multiple APN orthologs served as receptors for CCoV-HuPn-2018. (**A**) Transient expression of APN orthologs in 293T cells. The cell lysates were detected by western blot assay using an anti-C9 monoclonal antibody. β-Actin served as a loading control. (**B**) IP assay. The upper panel shows the input of APN protein with a C9 tag and RBD with an IgG Fc tag (RBD-Ig). The lower panel shows the APN pulled down by the RBD-Ig fusion protein. (**C–E**) Pseudotyped virus entry of CCoV-HuPn-2018 (**C**), TGEV (**D**), and HCoV-229E (**E**). 293T cells were transfected with the empty vector pcDNA3.1 or APN orthologs. At 48 h post-transfection, the cells were infected by the pseudotyped viruses CCoV-HuPn-2018 (CCoV-HuPn-2018pp), TGEV (TGEVpp), and TGEV (TGEVpp). At 24 h post-infection, pseudotype entry was determined by measuring luciferase activity in cell lysates. Luciferase signals obtained for particles bearing no envelope protein were used for normalization. Error bars reveal the standard deviation of the means from three biological repeats.

To provide an insight into the cross-species transmission of CCoV-HuPn-2018, we tested the receptor activities of APN in nine mammalian species, including humans, rhesus monkeys, porcines, canines, felines, Mexican free-tailed bats (*Tadarida brasiliensis*, Tb bats), rats (*Rattus norvegicus*), mice (*Mus musculus*), and rabbits (*Oryctolagus cuniculus*). These APN constructs expressed the APN proteins at a similar level in transfected 293T cells (Fig. 2A). Immunoprecipitation assays showed that APN orthologs from porcine, canine, feline, and Tb bat species, but not other species, were able to bind to the receptor binding domain (RBD) of the CCoV-HuPn-2018 S protein at a similarly efficient level (Fig. 2B). Consistently, only these four APN orthologs could efficiently support S protein-mediated VSV-based pseudotyped virus entry of CCoV-HuPn-2018 (Fig. 2C) and TGEV (Fig. 2D). As a control, the APN of humans, monkeys, and felines could efficiently support the pseudotyped virus entry of HCoV-229E (Fig. 2E).

### The N-glycosylation motif is critical for APNs to support CCoV-HuPn-2018 S protein-mediated entry

**FIG 3:**
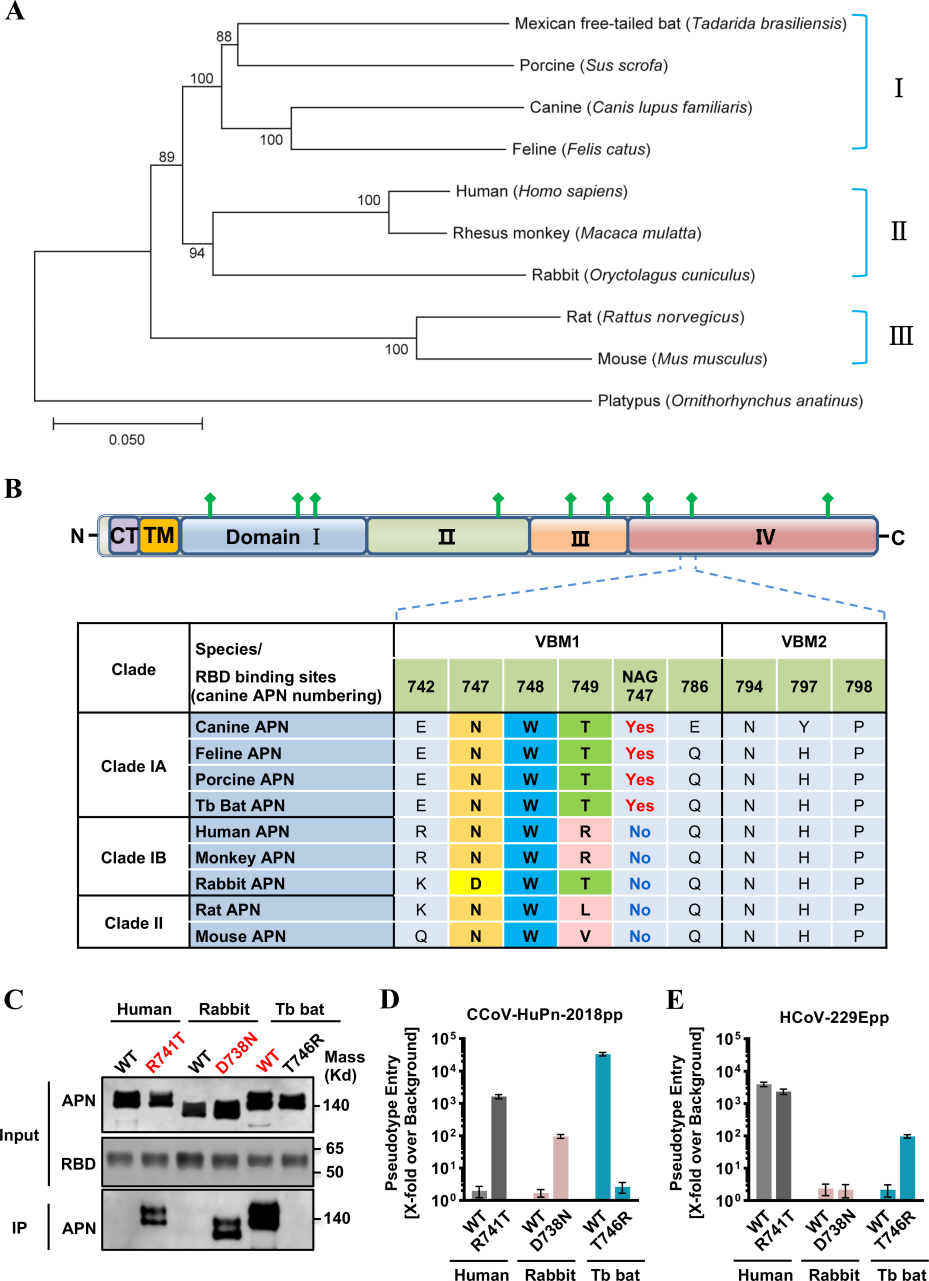
Glycosylation at residue 747 of APN is critical for its receptor activity. (**A**) Phylogenetic analysis of APN orthologs. Phylogenetic tree was constructed based on nucleotide sequences of the complete APN gene using the neighbor-joining method implemented in MEGA7.0.26 software. The percentage of replicate trees in which the associated taxa clustered together in the bootstrap test (1,000 replicates) is shown next to the branches. The tree was rooted by the APN of a platypus (*Ornithorhynchus anatinus*, GenBank No. XM_029051618.1). The animal species are shown on the right-hand side of the tree. (**B**) At the top is the domain organization of the canine APN. APN is a type II membrane glycoprotein that has a short N-terminal intracellular tail, a transmembrane domain, and a large C-terminal ectodomain. CT, cytoplasmic tail; TM, transmembrane domain; domains I, II, III, and IV are in blue, green, light orange, and light red, respectively. N-linked glycans are rendered as green protrusions. At the bottom is the sequence alignment of nine APNs focused on two VBMs and N747 glycan (canine APN numbering). One-letter codes are used for amino acids, and amino acids of the “N-W-T” motif are highlighted with different colors. (**C**) IP assay. The upper panel shows the input of wild-type APN proteins and APN mutants with a C9 tag and RBD with an IgG Fc tag (RBD-Ig). The lower panel shows the APN pulled down by the RBD-Ig fusion protein. (**D and E**) VSV-Luc-based pseudotyped virus entry. 293T cells were transfected with wild-type APN orthologs (human APN, rabbit APN, and bat APN) or APN mutants (human APN R741T glycan knockin mutant, rabbit APN D738N glycan knockin mutant, and bat APN T746R glycan knockout mutant). At 48 h post-transfection, the cells were infected by CCoV-HuPn-2018pp (**D**) or HCoV-229Epp (**E**). At 24 h post-infection, luciferase signals were measured, and pseudotype entry was normalized against the background (signal obtained for pseudotyped particles without viral envelope protein). Error bars reveal the standard deviation of the means from three biological repeats.

To understand the molecular basis of the differential receptor activities of these nine APN orthologs, we first examined their sequence variation. For this purpose, we constructed a phylogenetic tree based on the nucleotide sequences of APNs (Fig. 3A). Interestingly, the phylogenetic clustering of APNs is somewhat correlated with their ability to support the CCoV-HuPn-2018pp entry. Specifically, the four APNs (Tb bat, porcine, canine, and feline) that serve as functional receptors are placed in a single cluster, i.e., group I. APNs in groups II and III do not possess receptor activity. This correlation suggests that sequence variations between groups I and II/III may contribute to the observed differences in receptor activity.

Next, we focused the sequence variation on two virus-binding motifs (VBMs) of APN. According to the structure model of the canine APN (cAPN) ectodomain/CCoV-HuPn-2018 RBD complex (29), these two VBMs contain a total of seven residues (aa 742, 747–749, 786, 794, 797, 798, canine APN numbering) and one N-linked glycan at aa 747–749 (N-glycosylation motif, NWT) that directly contact CCoV-HuPn-2018 RBD (Fig. 3B). Upon close examination, we found that sequence variations at residues 742 and 747 distinguish group I from groups II and III. Previous studies have shown that the glycan at N747 (canine APN numbering) is critical for TGEV and CCoV-HuPn-2018 entry (29 – 31). To further investigate its importance for the receptor activity, we generated a Tb bat APN mutant (T746R, equivalent to canine APN position 749) with the N-glycosylation motif disrupted, a human APN mutant (R741T, equivalent to canine APN position 749), and a rabbit APN mutant (D738N, equivalent to canine APN position 747) with the N-glycosylation motif restored. Our results demonstrated that acquisition of the N-glycosylation signature (NWT) converted human and rabbit APNs to functional receptors for CCoV-HuPn-2018 and did not affect their receptor activity for HCoV-229E (Fig. 3C through E), whereas loss of the NWT abolished the receptor activity of the bat APN for CCoV-HuPn-2018 and surprisingly converted it to a functional receptor for HCoV-229E, indicating that the glycan at NWT is critical for APN receptor activity. It should be noted that acquisition of the N-glycosylation signature only partially converted human and rabbit APN mutants to an efficient receptor for CCoV-HuPn-2018, as the receptor activities of these two APN mutants are only 10% or 1% of those of bat APN, respectively (Fig. 3D), implying that other residues, e.g., aa 742, may also be important for the receptor activity.

Consistent with our finding above, homology-based structure modeling indicated that Tb bat APN maintained most residues in VBMs and the N-linked glycan that directly contacted CCoV-HuPn-2018 RBD. However, Tb bat APN_T746R_ lost N744 glycan, which disrupted the hydrogen bonds between RBD Q545 side chain and the first N-linked NAG and core fucose of Tb bat APN. In contrast, rabbit APN possessed two critical residue substitutions in VBM1, which resulted in the loss of the N-linked glycan and disrupted its interaction with CCoV-HuPn-2018 RBD. The mutation of D738N in rabbit APN restored the N-linked glycan and its associated interaction (Fig. S1).

### CCoV-HuPn-2018 S protein-mediated entry into multiple human cell lines in a human APN-independent manner

**FIG 4:**
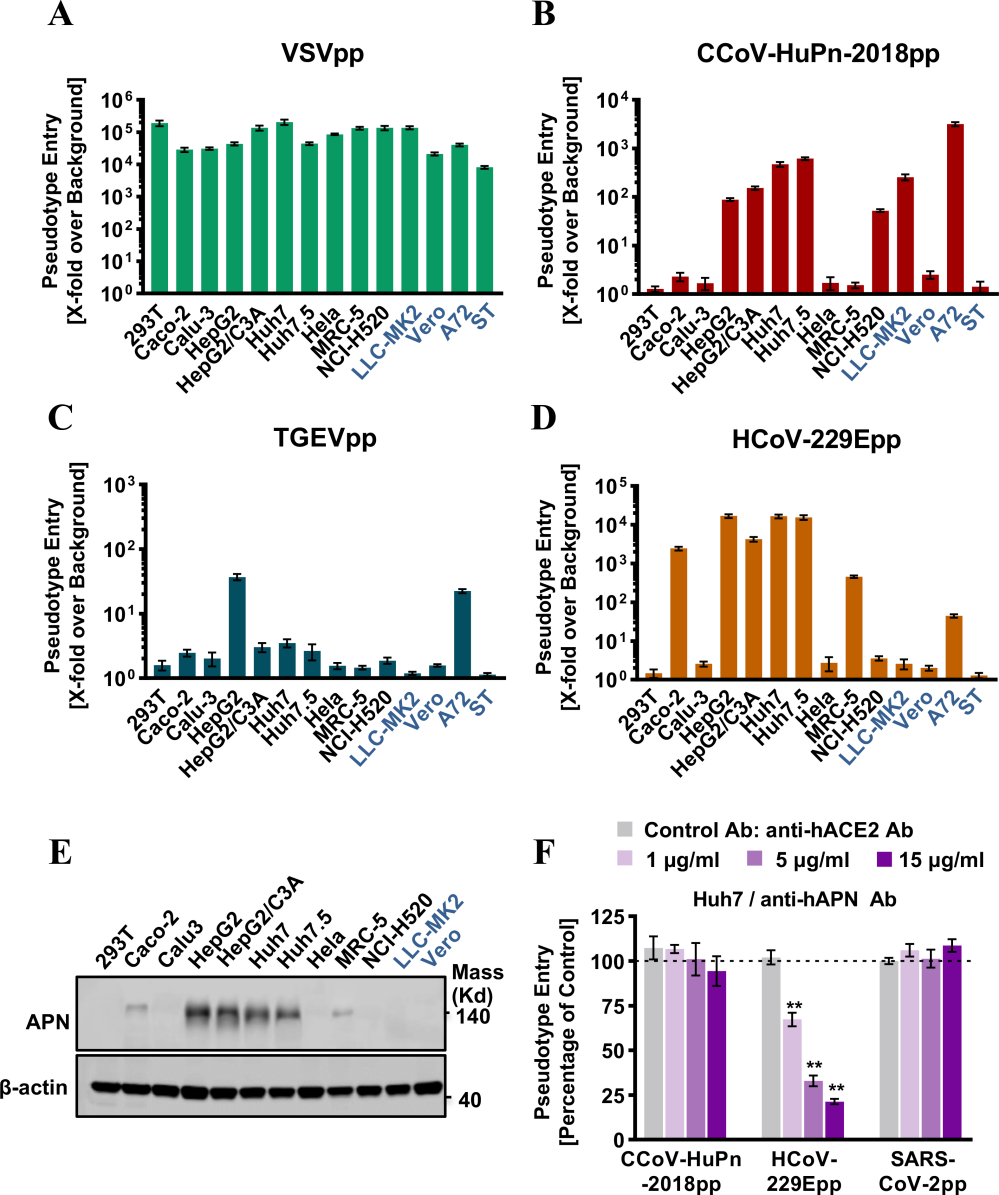
Entry of CCoV-HuPn-2018 into multiple human cell lines in a hAPN-independent manner. (A to D) Cell lines of human and other animal origins were infected with VSV-Gpp (**A**), CCoV-HuPn-2018pp (**B**), TGEV (**C**), or HCoV-229Epp (**D**). At 24 h post-infection, luciferase signals were measured, and pseudotype entry was normalized against the background (a luciferase signal was obtained for pseudotyped particles without viral envelope protein). Error bars reveal the standard deviation of the means from three biological repeats. Four animal-derived cell lines are shown in dark blue. (**E**) The levels of endogenous APN proteins were determined by Western blot assay using an anti-APN polyclonal antibody, and β-actin served as a loading control. (**F**) Entry inhibition assay by anti-hAPN antibody. Huh7 cells were pre-incubated with the indicated concentration of anti-hAPN antibody (or anti-hACE2 antibody as a control) for 1 h and then infected with CCoV-HuPn-2018pp in the presence of the indicated concentration of hACE2 antibody or hAPN antibody for another 3 h, and then the virus and antibodies were removed and replaced with fresh medium for further incubation. At 24 h post-infection, luciferase signals were measured, and pseudotype entry was normalized against the control antibody-treated cells. Error bars reveal the standard deviation of the means from three biological repeats. **, *P* < 0.001 compared to the level of the control antibody.

As shown in Fig. 2, human APN cannot be used as a receptor by CCoV-HuPn-2018. Then, how does CCoV-HuPn-2018 enter human cells? To answer this question, we first examined the susceptibility of 10 human cell lines and 4 animal-derived cell lines to infection by pseudotyped viruses of VSV (VSV-Gpp), CCoV-HuPn-2018 (CCoV-HuPn-2018pp), TGEV (TGEVpp), or HCoV-229E (HCoV-229Epp). Our results showed that all the cell lines could be readily infected by VSV-Gpp (Fig. 4A). However, the susceptibility profile of these cell lines was different between CCoV-HuPn-2018pp, TGEVpp, and HCoV-229Epp (Fig. 4B through D), suggesting different entry mechanisms used by these three alphacoronaviruses, although they all use APN as a receptor. Most strikingly, we found that four human hepatoma cell lines (HepG2, HepG2/C3A, Huh7, and Huh7.5) and one human lung cancer cell line (NCI-H520) are susceptible to CCoV-HuPn-2018pp infection, despite having a lower efficiency than canine cell line A72 (Fig. 4B). The endogenous APN expression levels in these human cell lines were positively proportional to the entry levels of HCoV-229Epp (Fig. 4D and E), which uses human APN as its functional receptor, but there was no correlation between the human APN levels and the entry levels of CCoV-HuPn-2018pp. For example, the entry level of CCoV-HuPn-2018pp was similar in HepG2 and LLC-MK2, despite HepG2 expressing much higher levels of APN (Fig. 4B and E). In addition, the entry level of CCoV-HuPn-2018pp in Huh7 cells was not affected by anti-hAPN antibodies (Fig. 4F). These findings suggest that CCoV-HuPn-2018 entered these human cell lines *via* a hAPN-independent pathway.

To exclude the possibility that CCoV-HuPn-2018 entered these human cell lines by using cellular receptors of other HCoVs, we used pseudotyped viruses including VSV-Gpp, CCoV-HuPn-2018pp, HCoV-229Epp, SARS-CoV-2pp, and MERS-CoVpp to infect 293T cells transfected by cAPN, hAPN, human ACE2 (hACE2, receptor for SARS-CoV-2, SARS-CoV, and HCoV-NL63), or human DPP4 (hDPP4, receptor for MERS-CoV), respectively. Our results showed that neither hACE2 nor hDPP4 supported the entry of CCoV-HuPn-2018pp (Fig. S2A) and anti-hACE2 antibodies did not block CCoV-HuPn-2018pp entry into Huh7 cells (Fig. S2B).

### Sialic acid contributes to CCoV-HuPn-2018 spike-mediated infection

**FIG 5:**
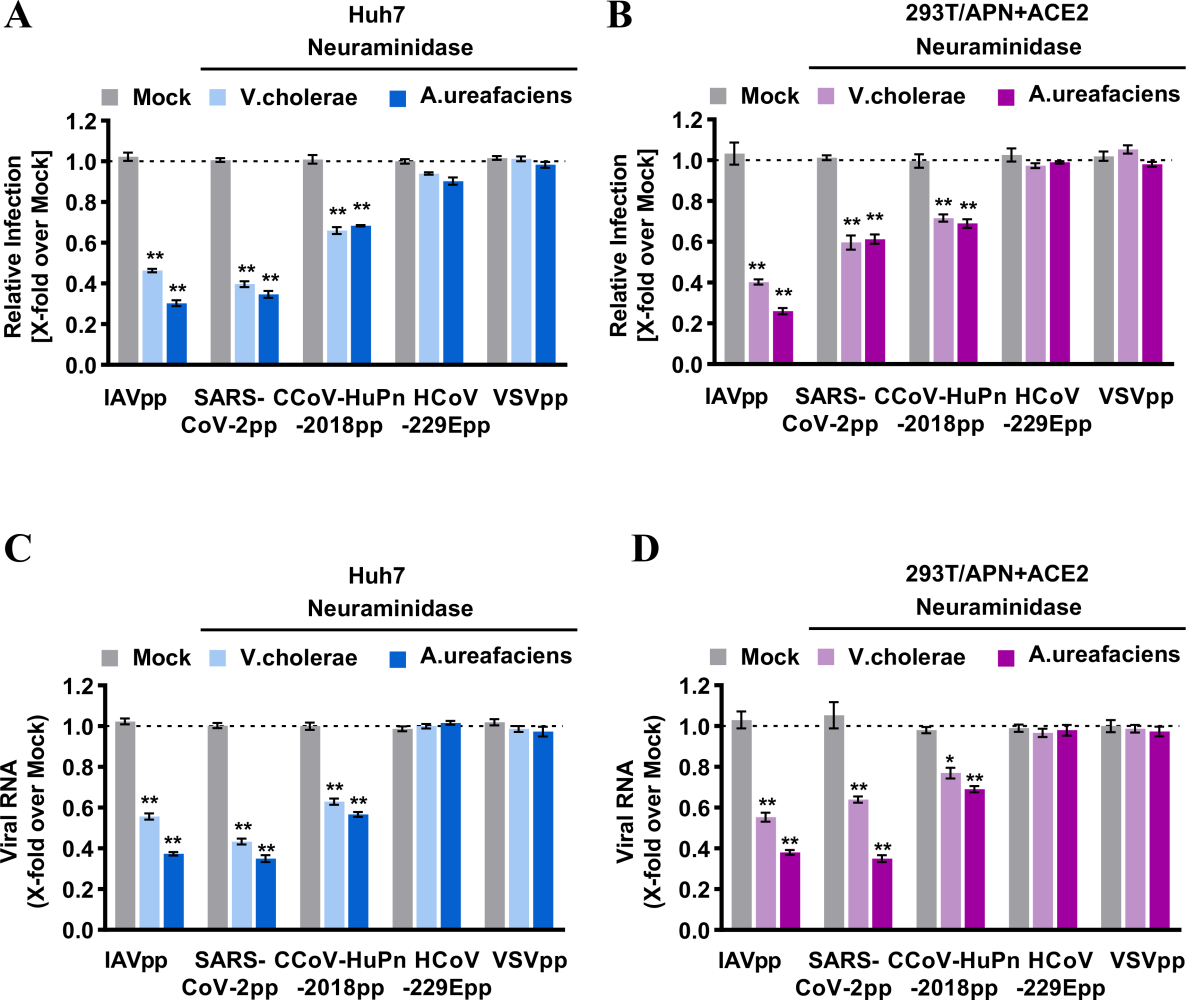
Sialic acid contributed to the entry of CCoV-HuPn-2018 into human cells. (**A and B**) Pseudotyped virus infection of neuraminidase-treated cells. Huh7 cells (**A**) or feline APN and human ACE2 cotransfected 293T cells (**B**) were pretreated with or without the indicated neuraminidases (neuraminidase from *Vibrio cholerae*, 40 mU/mL; from *Arthrobacter ureafaciens*, 100 mU/mL) at 37°C for 2 h, followed by infected with the indicated pseudoviruses at 37°C for 3 h. Then, the viruses and neuraminidases were removed and replaced with fresh medium for further incubation. At 24 h post-infection, cells were lysed, and luciferase activity was measured. Relative infection is the ratio of the luciferase activity in cells treated with neuraminidases over that in mock-treated cells. (**C and D**) Pseudotyped virus attachment on neuraminidase-treated cells. Huh7 cells (**C**) or feline APN and human ACE2 cotransfected 293T cells (**D**) were treated with or without neuraminidase for 2 h at 37°C, and then the cells were inoculated with the indicated HIV-based pseudotyped lentiviral particles at 4°C for 1 h. After extensive washing to remove unbound viruses, the cells were immediately harvested. The amounts of cell-associated viral RNA were quantified by qRT-PCR. Error bars reveal the standard deviation of the means from three biological repeats. *, *P* < 0.05; **, *P* < 0.001 compared to the level of mock-treated cells.

Besides known proteinaceous receptors, sugar moieties such as sialic acid can function as alternative receptors or attachment factors to support the entry of influenza A virus (IAV) and other CoVs, including TGEV (32), HCoV-OC43, and SARS-CoV-2 (27, 33). A recent study showed that the CCoV-HuPn-2018 spike protein interacts with sialic acid *via* its N-terminal A0 domain (29). To see if sialic acid mediated CCoV-HuPn-2018pp entry into those human cell lines, we first used neuraminidase from *Vibrio cholerae* or *Arthrobacter ureafaciens* to remove sialic acid from the surface of Huh7 cells, or APN and ACE2 cotransfected 293T cells, followed by infection with pseudotyped viruses. Our results showed that neuraminidase treatment significantly reduced the infection of CCoV-HuPn-2018pp, SARS-CoV-2pp, and IAVpp, but not HCoV-229Epp and VSVpp (Fig. 5A and B). In agreement with this finding, the amounts of pseudotyped viral particles attached to the cell surface were significantly reduced by the neuraminidase treatment for CCoV-HuPn-2018pp, SARS-CoV-2pp and IAVpp, but not for HCoV-229Epp and VSV-Gpp (Fig. 5C and D). These results suggest that CCoV-HuPn-2018 exploits sialic acid as a receptor or attachment factor to mediate its entry into human cells.

### Trypsin treatment and TMPRSS2 do not promote CCoV-HuPn-2018 spike-mediated entry into target cells

**FIG 6:**
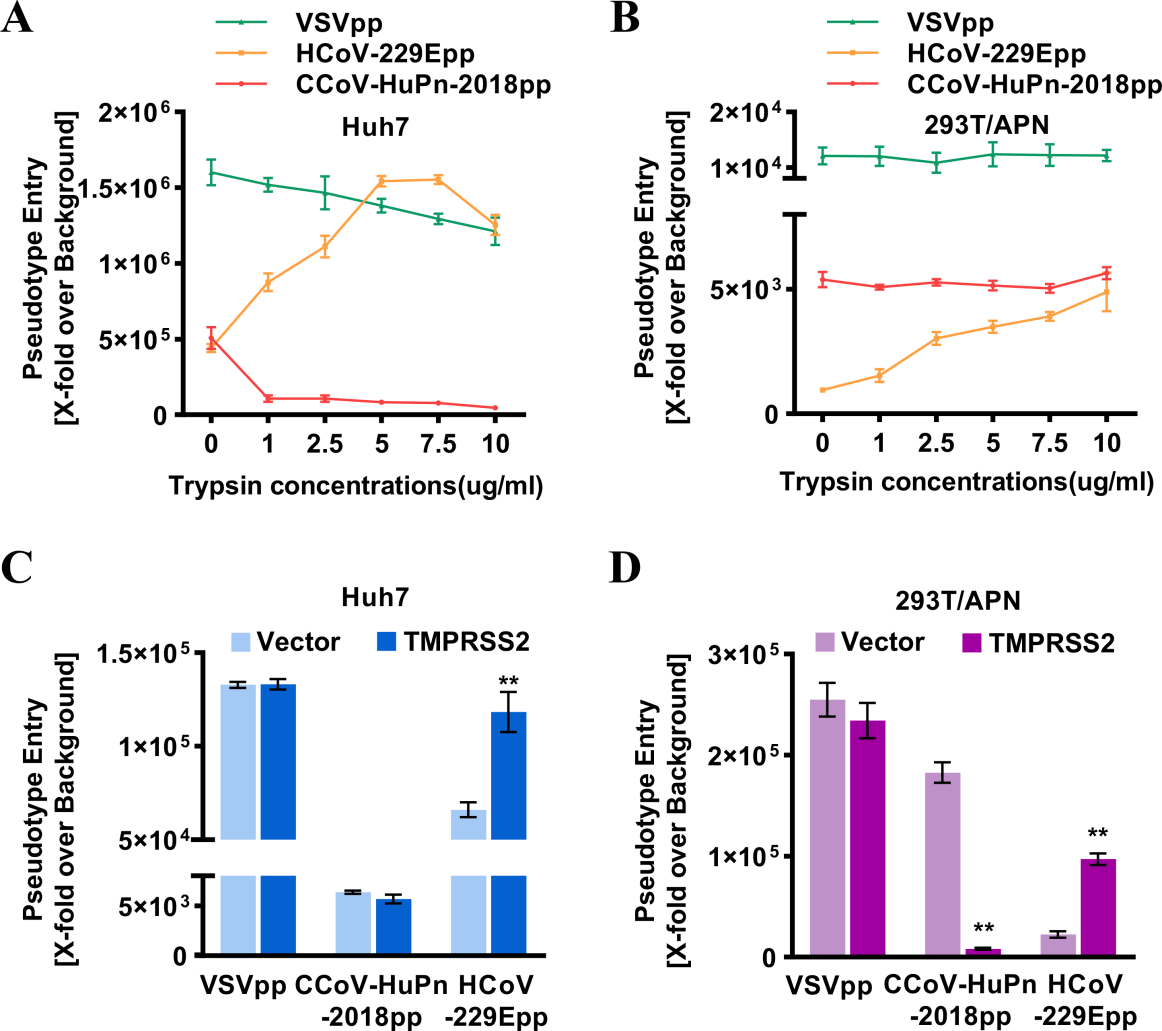
Trypsin and TMPRSS2 did not promote the cell entry of CCoV-HuPn-2018. (**A and B**) Huh7 cells (**A**) or fAPN transiently transfected 293T cells (**B**) were infected with CCoV-HuPn-2018pp after centrifuging at 4°C at 3,500 × *g* for 35 min. Infected cells were treated with the indicated concentrations of TPCK-trypsin or DMEM without FBS at 37°C for 13 min. Luciferase activities were measured at 24 h post-infection, and pseudotype entry was normalized against pseudotyped particles without viral envelope protein. (**C and D**) Huh7 cells transiently transfected with pCAGGS vector or TMPRSS2 (**C**), 293T cells transiently cotransfected with fAPN and pCAGGS, or fAPN and TMPRSS2 (**D**) were infected with the indicated pseudotyped viruses. At 24 h post-infection, luciferase activities were measured, and pseudotype entry was normalized against pseudotyped particles without viral envelope protein. Error bars reveal the standard deviation of the means from three biological repeats. **, *P* < 0.001 compared to the level of mock-treated cells.

Proteolytic cleavage of CoV S proteins is a critical priming step for membrane fusion. To investigate the role of cell surface proteases on CCoV-HuPn-2018pp entry, Huh7 and 293T cells expressing feline APN (fAPN) were either treated with exogenous trypsin or transfected with a plasmid expressing TMPRSS2, followed by infection with CCoV-HuPn-2018pp, with VSVpp and HCoV-229Epp as negative and positive controls, respectively. As anticipated, both trypsin treatment and TMPRSS2 expression significantly enhanced the infection of HCoV-229Epp, but not VSVpp, in Huh7- and fAPN-expressing 293T cells. However, trypsin treatment or expression of TMPRSS2 did not enhance the entry level of CCoV-HuPn-2018pp in either Huh7- or fAPN-expressing 293T cells (Fig. 6A through D).

### Endosomal protease(s) are required for CCoV-HuPn-2018 spike-mediated infection

**FIG 7:**
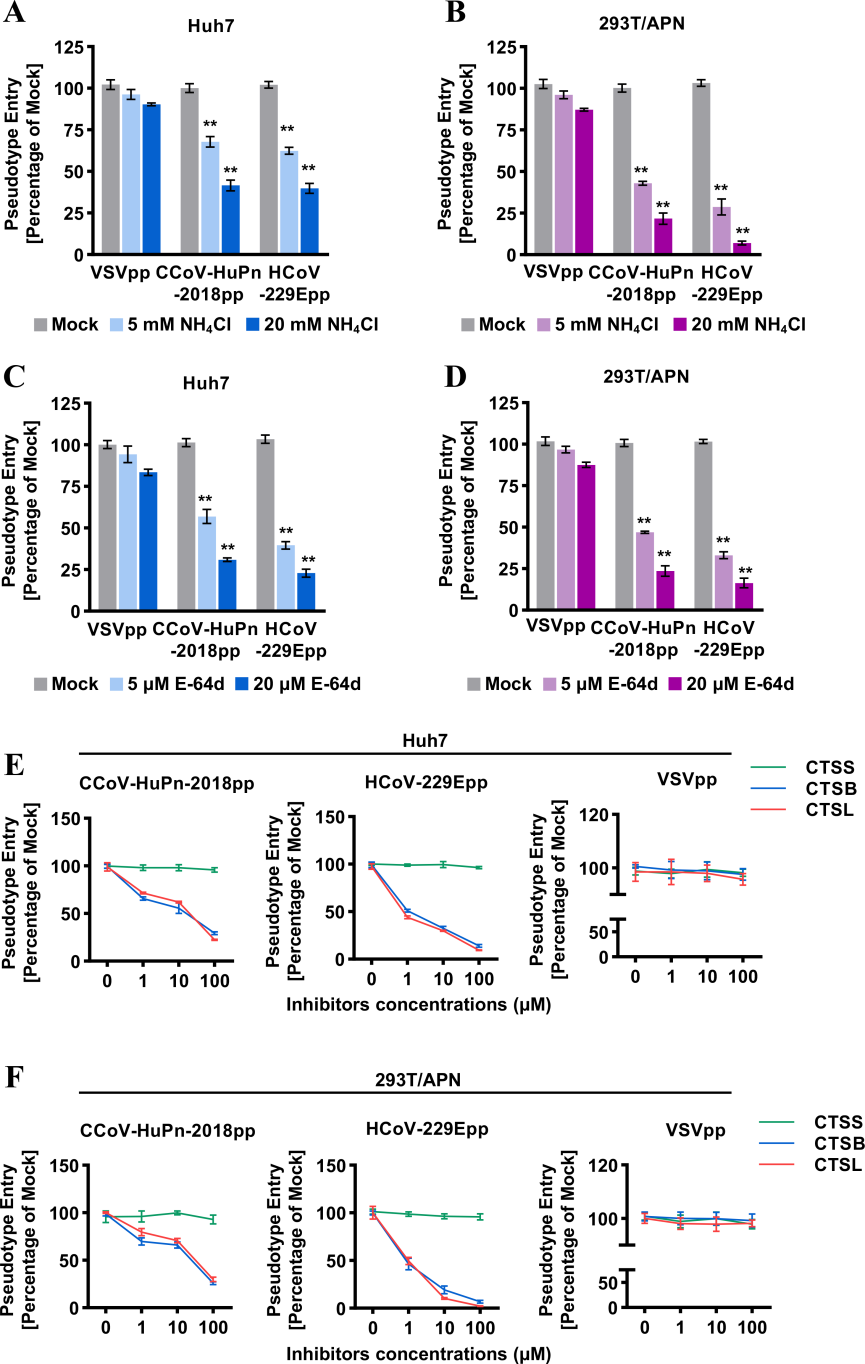
Endosomal protease(s) are required for CCoV-HuPn-2018 spike-mediated infection. (A to F) Huh7 cells (A, C, and E) or fAPN transiently transfected 293T cells (B, D, and F) were infected with CCoV-HuPn-2018pp in the absence (mock) or presence of the indicated concentrations of NH_4_Cl (**A and B**), E-64d (**C and D**), or different cathepsin inhibitors (**E and F**). Luciferase activities were determined 24 h post-infection. Pseudotype entry is the ratio of the luciferase activity in cells treated with inhibitors over that in mock-treated cells. Error bars reveal the standard deviation of the means from three biological repeats. **, *P* < 0.001 compared to the level of mock-treated cells.

Coronaviruses enter host cells through membrane fusion at the plasma membrane in a pH-independent manner or in endosomal compartments in a pH-dependent manner. To investigate the entry route of CCoV-HuPn-2018pp, Huh7 cells or fAPN-expressing 293T cells were treated with NH_4_Cl, an endosomal acidification inhibitor, or E-64d, an inhibitor of endosomal cathepsins L, B, H, etc. (34), and then infected by CCoV-HuPn-2018pp or HCoV-229Epp. Our results showed that the entry of CCoV-HuPn-2018pp and HCoV-229Epp was significantly inhibited by NH_4_Cl (Fig. 7A and B) and E-64d (Fig. 7C and D) in a dose-dependent manner. To further elucidate which cathepsin is involved in proteolytic cleavage of S protein and membrane fusion, the effects of inhibitors of cathepsin B (CTSB), cathepsin L (CTSL), and cathepsin S (CTSS) on CCoV-HuPn-2018pp infection were examined. We found that inhibitors of CTSB and CTSL, but not an inhibitor of CTSS, efficiently inhibited the entry of CCoV-HuPn-2018pp and HCoV-229Epp in Huh7 cells and fAPN-expressing 293T cells (Fig. 7E and F). As a negative control, the entry of VSVpp was not affected by any of these cathepsin inhibitors. These results suggest that endosomal proteases cathepsin L and B are required for CCoV-HuPn-2018 spike-mediated entry in the low pH endosomal compartments.

### IFITMs and LY6E restrict CCoV-HuPn-2018 spike-mediated viral entry

**FIG 8:**
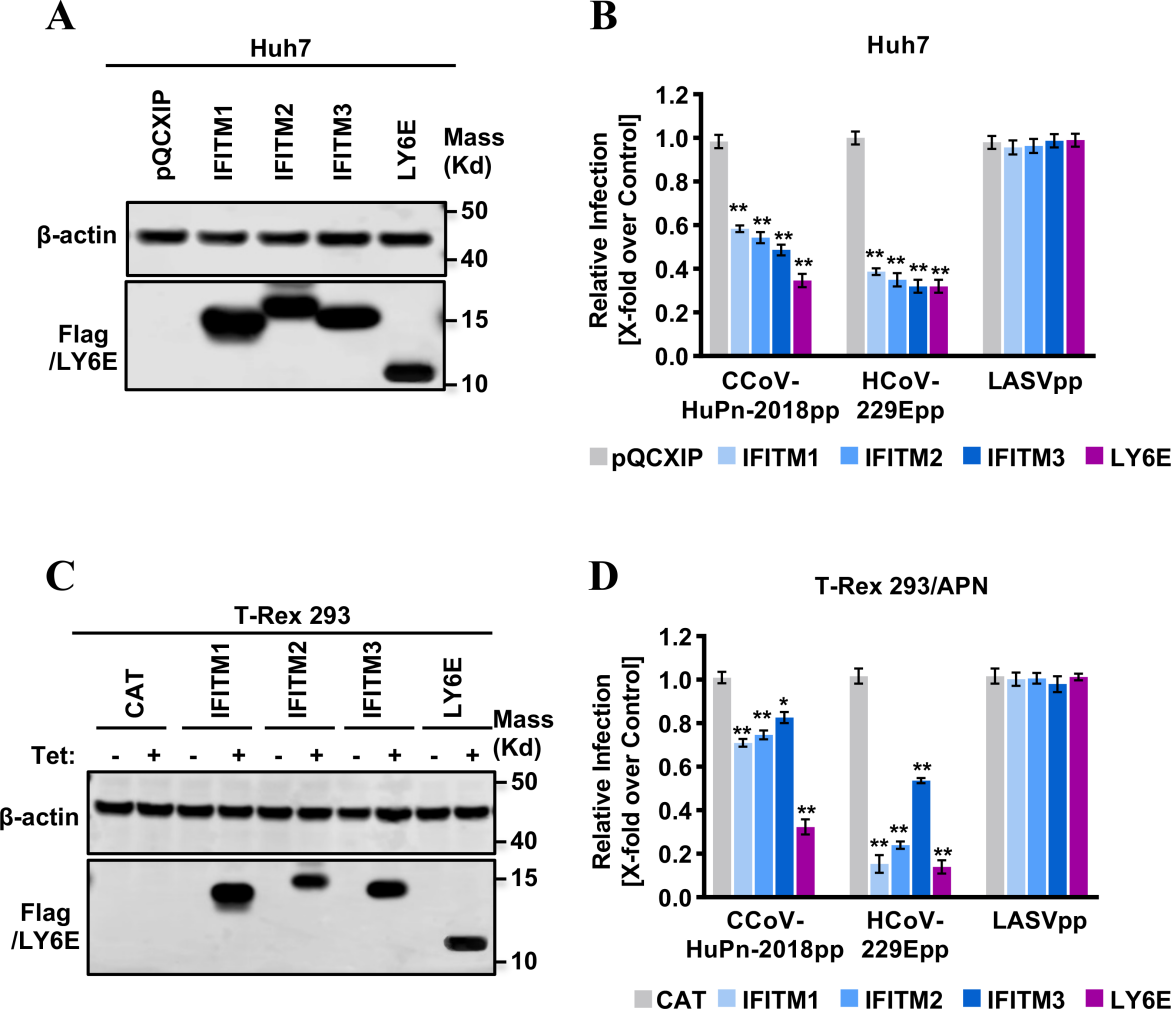
Inhibition of CCoV-HuPn-2018 entry by IFITMs and LY6E. (**A**) The expression of LY6E and FLAG-tagged IFITMs in Huh7-derived stable cell lines was detected by a Western blot assay using a rabbit polyclonal antibody against LY6E and a monoclonal antibody against the FLAG tag. β-Actin served as a loading control. (**B**) Inhibition of the entry of CCoV-HuPn-2018pp by IFITMs and LY6E. Huh7 cells stably expressing N-terminally FLAG-tagged human IFITM proteins, LY6E protein, or transduced with an empty vector (pQCXIP) were infected with CCoV-HuPn-2018pp and other indicated pseudoviral particles. Luciferase activities were determined 24 h post-infection. Relative infection is the ratio of luciferase activity of cells expressing the indicated IFITM protein or LY6E protein over that of cells transduced with an empty vector. (**C**) The expression of a control protein CAT, LY6E protein, or FLAG-tagged IFITM proteins in Flp-In T-Rex 293 cells cultured in the absence or presence of 2 µg/mL of tet for 24 h was detected by a Western blot assay using a rabbit polyclonal antibody against LY6E and a monoclonal antibody against the FLAG tag. β-Actin served as a loading control. (**D**) Inhibition of APN-dependent entry of CCoV-HuPn-2018pp by IFITMs and LY6E. Flp-In T-Rex 293 cells expressing CAT, the indicated IFITM protein, or the LY6E protein were transfected with feline APN, cultured in the presence or absence of tet for 24 h, and then infected with CCoV-HuPn-2018pp and other indicated pseudoviral particles. Luciferase activities were determined 24 h post-infection. Relative infection efficiency is the ratio of luciferase activity in cells cultured in the presence of tet over that in cells cultured in the absence of tet. Error bars reveal the standard deviation of the means from three biological repeats. *, *P* < 0.05; **, *P* < 0.001 compared to the level of empty vector-transduced cells.

The family of IFN-induced transmembrane (IFITM) proteins (IFITM1, IFITM2, and IFITM3) and lymphocyte antigen 6 family member E (LY6E) have been demonstrated by others and us to either restrict or promote cell entry of HCoVs and play important roles in coronaviral pathogenesis (35 – 39). Here, we examined the effects of three human IFITM proteins (IFITM1, IFITM2, IFITM3) and the LY6E protein on the APN-dependent and -independent entry of CCoV-HuPn-2018pp in Huh7 cells and Flp-In T-Rex 293-derived cell lines transfected with a plasmid expressing fAPN (Fig. 8A and C), with pseudotyped Lassa fever virus (LASVpp) and HCoV-229Epp as negative and positive controls, respectively. Our results showed that overexpression of IFITM1, IFITM2, IFITM3, or LY6E significantly inhibited the infection of CCoV-HuPn-2018pp and HCoV-229Epp (Fig. 8B and D). As a negative control, the infection of LASVpp was not affected by any of these proteins.

## DISCUSSION

In this study, we examined the receptor activity of nine APN orthologs, critical residues for receptor usage, cell tropism, other host factors, and restriction factors for cell entry of CCoV-HuPn-2018. Results obtained from this study help us understand the underlying mechanism of cell entry, assess the pathologic potential and risk of human-to-human transmission of this potential human CoV, and shed light on the interspecies transmission and natural reservoir.

During our investigation of the receptor usage of CCoV-HuPn-2018, another research group recently reported that this virus uses cAPN, fAPN, and porcine APN (pAPN), but not hAPN, as functional receptors for cell entry (29). This was confirmed by our results (Fig. 2 and 4F). Given that CCoV-HuPn-2018 is essentially a CCoV that is closely related to other CCoV isolates, FCoVs, and TGEV (Fig. 1), it is not surprising that cAPN, fAPN, and pAPN are the efficient receptors. However, it is surprising to find that CCoV-HuPn-2018 does not use hAPN as a receptor. Further study showed that this is mainly due to the lack of an N-glycosylation site at residues 747–749 of hAPN (Fig. 3C and D). Then, how does this virus enter human cells?

One possibility is that the APN of some people may possess the N-glycosylation site at residues 747–749, e.g., a threonine, instead of an arginine, at residue 749, which will render these people susceptible to the CCoV-HuPn-2018 infection. However, such people must be extremely rare, as no T749 was found in the gnomAD database, which includes ~125,748 whole exome sequences of humans.

The other possibility is that this virus may use other host factors to enter human cells. First, we excluded the possibility that it uses other known receptors for HCoVs, i.e., hACE2 and hDPP4 (Fig. S2). Second, we found that CCoV-HuPn-2018pp could infect five human cancer cell lines with varying levels of efficiency (Fig. 4B) and confirmed that this infection was not mediated by hAPN as the endogenous hAPN levels in these human cell lines were not correlated with the CCoV-HuPn-2018pp entry levels (Fig. 4E) and anti-hAPN antibodies did not block the entry of CCoV-HuPn-2018pp (Fig. 4F). Third, we found that neuraminidase treatment could significantly reduce the virus’s binding to the surface of and entry into Huh7 cells and APN-transfected 293T cells in a dose-dependent manner (Fig. 5), suggesting that sialic acid may mediate the APN-dependent and -independent entry of CCoV-HuPn-2018pp into these human cells. This conclusion is in line with a recent study showing that the CCoV-HuPn-2018 S protein interacts with sialic acid *via* its N-terminal A0 domain with homology to TGEV (29, 40). Sialic acid can also bind to the N-terminal domain of S proteins of several CoVs, including TGEV, infectious bronchitis virus, bovine coronavirus, HCoV-OC43, and MERS-CoV, and promote their infection (12). It should be noted that, in contrast to its role in influenza A virus infection, where it serves as a receptor, sialic acid is generally believed to be only an attachment factor for coronaviruses, especially for those CoVs with specific protein receptors, e.g., TGEV (12). Therefore, its ability to mediate CCoV-HuPn-2018pp entry into host cells may be limited.

Proteolytic cleavage of CoV S proteins by host protases, e.g., TMPRSS2, is a critical priming step for membrane fusion. It modulates the pathogenicity, cell and tissue tropism, evasion of intrinsic immunity, and extended host range of coronaviruses (12). For example, SARS-CoV-2 possesses a furin-like cleavage site at this S1/S2 boundary of the S protein and exploits TMPRSS2 to enhance virus entry, evade innate antiviral defenses, and facilitate transmission (41). For CCoV-HuPn-2018, we found that the cell surface proteases trypsin and TMPRSS2 did not enhance the pseudovirus entry (Fig. 6). Interestingly, TMPRSS2 expression significantly reduced CCoV-HuPn-2018pp infection in 293/APN cells but not Huh7 cells, and the underlying mechanism needs further investigation. Sequence analysis shows that the S1/S2 boundary of the CCoV-HuPn-2018 S protein does not harbor the cleavage motif for trypsin, TMPRSS2, or furin (Fig. S3).

Collectively, our results suggest that the current risk of CCoV-HuPn-2018 infection in humans is still low as it has not yet evolved to become an efficient human pathogen, primarily due to the inability to use hAPN as a receptor. Although occasional spillover events occurred, possibly *via* sialic acid attachment, the level of virus entry may be low and is further limited by the virus’s inability to be cleaved by host proteases trypsin and TMPRSS2. This may reduce the likelihood for CCoV-HuPn-2018 to undergo sustained human-to-human transmission. In addition, a previous study has shown that plasma from HCoV-229E-infected patients could a provide certain level of protection from CCoV-HuPn-2018 infection (29). Since HCoV-229E is constantly circulating in humans, many people may already have been infected and possess pre-existing adaptive immunity against CCoV-HuPn-2018 infection. This further ameliorates the risk of human-to-human transmission of CCoV-HuPn-2018. Still, given that CoVs are prone to sequence variation and frequency recombination, surveillance of the incidence of CCoV-HuPn-2018 in humans is needed. Of note, there is one limitation: our data collected from pseudoviruses may not fully display the cellular entry of authentic CCoV-HuPn-2018, which should be further validated with authentic viruses in future studies.

One interesting finding of this study is that, among the nine APN orthologs examined, in addition to the APNs of canine, feline, and porcine, the Tb bat APN could serve as an efficient receptor for CCoV-HuPn-2018 (Fig. 2). We further showed that the N-glycosylation site at N747-W748-T749 of APN was the key determinant for the receptor activity, as the bat APN mutant without this glycosylation site nearly completely lost its receptor activity for CCoV-HuPn-2018 (Fig. 3C and D; Fig. S1). We found that APNs from multiple bat species also possess this N-linked glycosylation site (Fig. S4); therefore, all these bat APNs may be efficient receptors for CCoV-HuPn-2018. This raises the possibility that bats may be an alternative host epidemiologically important for the transmission of CCoV-HuPn-2018, as well as for other emerging HCoVs, e.g., SARS-CoV, SARS-CoV-2, and MERS-CoV (5, 6, 42). These findings warrant investigating the occurrence and APN receptor usage of CCoV-HuPn-2018-like bat coronaviruses (BtCoVs).

In addition to the above-mentioned results, we first demonstrated that endosomal proteases cathepsin L and B are required for the entry of CCoV-HuPn-2018 in a pH-dependent manner (Fig. 7), and that it is the overexpression of IFITMs and LY6E that restricts the entry of CCoV-HuPn-2018pp (Fig. 8). These findings help us understand the underlying mechanism of cell entry in this potential human CoV.

## MATERIALS AND METHODS

### Cell cultures

293T (human, kidney), Huh7 (human, liver), Huh7.5 (human, liver), Hela (human, cervix), and Vero (African green monkey, kidney) cells were cultured in Dulbecco’s modified Eagle’s medium (DMEM; Gibco) supplemented with 10% fetal bovine serum (FBS), 110 mg/L sodium pyruvate, and 4.5 g/L D-glucose. Caco-2 (human, colon), Calu-3 (human, lung), MRC-5 (human, lung), and ST (*Sus scrofa* pig, testis fibroblast) cells were incubated in Eagle’s Minimal Essential Medium (ATCC, Cat. No. 30-2003). Human hepatoma cell lines HepG2 and C3A, a subclone of HepG2 (ATCC, Cat. No. HB-8065), were purchased from the ATCC and cultured in DMEM/F12 medium supplemented with 10% heat-inactivated FBS (Invitrogen). The human lung cancer cell line NCI-H520 was grown in RPMI-1640 medium (ATCC, Cat. No. 30-2001) supplemented with 10% FBS, penicillin G, and streptomycin. LLC-MK2 cells were cultured in minimal essential medium (MEM), which was prepared by mixing Hanks MEM (Invitrogen, Cat. No. 11575-032) and Earle’s MEM (Invitrogen, Cat. No. 11095-080) in a 2:1 ratio and supplemented with 10% heat-inactivated FBS (Invitrogen). A72 (*Canis familiaris*, tumor fibroblast) was incubated in Leibovitz’s L-15 Medium (ATCC, Cat. No. 30-2008) supplemented with 10% FBS. Flp-In T-Rex293 cells were purchased from Invitrogen and maintained in DMEM supplemented with 10% FBS, 10 µg/mL blasticidin (Invitrogen), and 100 µg/mL Zeocin (Invivogen) (43). Flp-In T-Rex293-derived cell lines expressing IFITM1, IFITM2, IFITM3, or LY6E were cultured in DMEM supplemented with 10% FBS, 5 µg/mL blasticidin, and 250 µg/mL hygromycin. All cell lines were incubated at 37°C and 5% CO_2_ in a humidified atmosphere.

### Antibodies

A polyclonal antibody against human ACE2 and a human APN polyclonal antibody were purchased from R&D Systems (Cat. Nos. AF933 and AF3815, respectively). A monoclonal antibody against the C9 tag was purchased from Santa Cruz (Cat. No. sc-57432). Monoclonal antibodies against the FLAG tag (anti-FLAG M2) and β-actin were purchased from Sigma (Cat. Nos. F1804 and A2228, respectively). Rabbit polyclonal antibodies against APN and human LY6E were purchased from Proteintech (Cat. Nos. 14553-1-AP and 22144-1-AP, respectively).

### Plasmids

For the construction of receptor expression plasmids, ACE2, APN, and DPP4 cDNA clones were obtained from Origene and cloned into a pcDNA3 vector (Invitrogen) to yield plasmids pcDNA3/ACE2, pcDNA3/APN, and pcDNA3/DPP4, respectively (44). APNs of humans, rhesus monkeys, porcines, canines, felines, Tb bats, rats, mice, and rabbita were cloned into the Nhe I/Xba I-digested pcDNA3.1-N-myc-C9 vector. The APN protein expressed from this vector has a C9 tag at the C-terminus. The wild-type full-length human APN and rabbit APN encoding plasmids were used as templates to introduce a glycosylation motif at amino acid positions 739–741 (NWR to NWT) to generate human APN R741T glycan knockin mutants and rabbit APN D738N glycan knockin mutants by overlapping PCR-based methods as previously described (45). A full-length wild-type bat APN-encoding plasmid was used as a template to knockout the glycan at positions 744–746 (NWT to NWR) to generate a bat APN T746R glycan knockout mutant with the same strategy. All the resulting plasmids were sequenced to verify the desired mutation(s).

For the construction of plasmids expressing S and RBD coronaviruses, plasmids expressing VSV G protein, Lassa fever virus (LASV) glycoprotein (GP), H1N1 IAV (A/WSN/33) hemagglutinin (HA) and neuraminidase (NA), TGEV spike glycoprotein, HCoV-229E spike protein, and MERS-CoV spike protein were described previously (46, 47). The acid amino sequence of the CCoV-HuPn-2018 S protein was retrieved from the National Center for Biotechnology Information database (GenBank No. QVL91811.1). According to the method described by Babcock et al. (48), the codon-optimized S gene was synthesized and cloned into the pSecTag2 vector. To produce soluble RBD-Ig fusion proteins, the RBD (aa 526–669) of CCoV-HuPn-2018 was cloned into a soluble protein expression vector, pSecTag2/Hygro-Ig vector, which contains a human IgG Fc fragment and a mouse Ig k-chain leader sequence (45). The protein (RBD-Ig) expressed is soluble and has a human IgG-Fc tag.

Plasmid pNL4-3.Luc.R-E− was obtained through the NIH AIDS Research and Reference Reagent Program (49, 50). The cDNA molecule of LY6E was purchased from OriGene (Cat. No. RC211373) and cloned into the pcDNA5/FRT-derived vector as described previously (51). LY6E and N-terminally FLAG-tagged human IFITM1, IFITM2, and IFITM3 were cloned into the pQCXIP vector (Clontech) between the NotI and BamHI sites as previously described (35, 51). pcDNA5/FRT-derived plasmids expressing chloramphenicol acetyltransferase (CAT) and N-terminally FLAG-tagged human IFITM1, IFITM2, and IFITM3 were reported previously (43, 52, 53).

### Establishment of cell lines expressing IFITM or LY6E proteins

Huh7 cells in each well of 6-well plates were incubated with 2 mL of Opti-MEM medium containing pseudotyped retroviruses and centrifuged at 20°C for 30 min at 4,000 × *g*. At 48 h post-transduction, the cells were cultured with medium containing 2 µg/mL of puromycin for 2 weeks. The antibiotic-resistant cells were pooled and expanded into cell lines stably expressing human IFITM or LY6E proteins (44). Flp-In T-Rex 293-derived cell lines expressing IFITMs or LY6E proteins in a tetracycline (tet)-inducible manner were established as previously described (43, 53).

### Western blot assay

Cell monolayers were washed once with phosphate-buffered saline (PBS) and lysed with 1× Laemmli buffer. An aliquot of cell lysate was separated on a NuPAGE Novex 4 to 12% Bis-Tris Gel (Invitrogen) and electrophoretically transferred onto a nitrocellulose membrane (Invitrogen). A polyvinylidene difluoride membrane containing the proteins transferred from an SDS-PAGE gel was blocked with blocking buffer [5% nonfat dry milk in Tris-buffered saline (TBS)] for 1 h at room temperature and probed with a primary antibody overnight at 4°C. The blot was washed three times with washing buffer (0.05% Tween-20 in TBS), followed by incubation with the secondary antibody for 1 h at room temperature. After three washes, the proteins bound to antibodies were visualized with IRDye secondary antibodies and imaged with a Li-Cor Odyssey system (Li-Cor Biotechnology).

### Immunoprecipitation assay

The association between Ig-fused RBD protein and APN protein with a C9 tag was measured by immunoprecipitation (IP) according to a previously described method (45). Briefly, 293T cells were transfected with plasmids encoding APN orthologs by Lipofectamine 2000 (Invitrogen). At 48 h post-transfection, the transfected 293T cells were harvested and lysed in PBS buffer containing 0.3% *n*-decyl-β-D-maltopyranoside (Anatrace) and protease inhibitors (Roche). Cell lysates were incubated with Protein A/G Plus Agarose (Santa Cruz, sc-2003) together with 4 µg of RBD-Ig. Protein A/G Agarose-treated cells were washed three times in TBS-1% Triton X-100, resolved by SDS-PAGE, and detected by western blot using an anti-C9 monoclonal antibody.

### Production of pseudotyped viruses

VSV-based pseudotyped viruses were generated according to a previously published protocol (54). In brief, 293T cells in 6-well plates were transfected by corresponding plasmids encoding for: VSV G protein (or no viral protein, control), LASV GP, H1N1 IAV (A/WSN/33) HA and NA, TGEV S protein, HCoV-229E S protein, MERS-CoV S protein, SARS-CoV-2 S protein, or CCoV-HuPn-2018 S protein by Lipofectamine 2000 (Invitrogen). After an incubation period of 6 h at 37°C, DMEM supplemented with 10% FBS was added and transduced with a replication-deficient VSV vector that contains expression cassettes for firefly luciferase instead of the VSV-G open reading frame, VSV*ΔG-fLuc (54). On the next day, the virus inoculum was removed, and the cells were washed twice with DMEM to minimize background from the parental viruses. Supernatants containing pseudotyped viral particles were collected at 48 and 72 h post-transfection, mixed and centrifuged at 3,000 × *g* for 5 min to remove cellular debris, and then filtered through a 0.45-µm pore-size PES syringe filter (Millipore) and stored at −80°C for future use.

For packaging of HIV-based pseudotyped retroviruses, the various viral envelope protein pseudotyped lentiviruses bearing luciferase reporter genes were packaged as reported previously (44). Briefly, 293T cells in six-well plates were cotransfected with 3 µg of pNL4.3.Luc.R-E^−^ and 1 µg of plasmid expressing a virus envelope protein by using Lipofectamine 2000 (Invitrogen). At 24 h post-transfection, the cells were replenished with 4 mL complete DMEM. Supernatants containing pseudotyped viral particles were collected at 48 and 72 h post-transfection, mixed and centrifuged at 3,000 × *g* for 5 min to remove cellular debris, and then filtered through a 0.45-µm pore-size PES syringe filter (Millipore) and stored at −80°C for future use.

### Pseudotyped virus entry assay

For experiments addressing receptor usage, each well of 293T cells in a 96-well plate was transfected with 0.1 µg of an empty vector plasmid or plasmids encoding for different viral receptors by using Lipofectamine 2000 (Invitrogen). At 48 h post-transfection, the cells were infected with the desired pseudotyped viral particles at 37°C for 3 h and then replenished with fresh medium for further incubation. At 24 h post-infection (hpi), the medium was removed, and the cells were lysed with 30 µL/well of 1× cell lysis buffer (Promega) for 15 min, followed by adding 50 µL/well of luciferase substrate (Promega). The firefly luciferase activities were measured by luminometry in a TopCounter instrument (PerkinElmer) (44).

For the cell tropism assay of pseudotyped viruses, cells of different origins were inoculated in black-walled 96-well plates overnight at 37°C and then infected with the desired pseudotyped viruses. The luciferase activities were measured at 24 hpi.

For pseudotyped virus neutralization assays with polyclonal antibodies, Huh7 cells in 96-well plates were preincubated with the indicated concentrations of human ACE2 antibody or human APN antibody at 37°C for 1 h, followed by infection with pseudotyped viral particles of CCoV-HuPn-2018pp, HCoV-229Epp, or SARS-CoV-2pp in the presence of the indicated concentrations of human ACE2 antibody or human APN antibody for another 3 h. Then, the viruses and antibodies were removed and replaced with fresh medium for further incubation. At 24 hpi, luciferase activities were measured and normalized to the level of the control antibody for each pseudotyped virus. For each experiment, four wells were tested in a single experiment, and at least three repeat experiments were carried out.

Flp-In T-Rex 293-derived IFITM- or LY6E-expressing cell lines were seeded into 96-well plates with black walls and clear bottoms and transfected with plasmids encoding feline APN to express viral receptors. For Huh7-derived IFITM- or LY6E-expressing cell lines, cells were seeded into black-wall 96-well plates. The cells were infected with the desired HIV-based pseudotyped lentiviral particles 48 h post-transfection, and luciferase activities were measured at 48 hpi. For each experiment, four wells were tested in a single experiment, and at least three repeat experiments were carried out.

### Inhibitor treatment assays

For the neuraminidase treatment assay, Huh7 cells or APN and ACE2 cotransfected 293T cells were pretreated with or without the indicated concentrations of neuraminidase from *Vibrio cholerae* (Roche, Cat. No. 11080725001, 40 mU/mL) or *Arthrobacter ureafaciens* (Roche, Cat. No. 10269611001, 100 mU/mL) at 37°C for 2 h, followed by infection with pseudotyped viral particles of IAVpp, SARS-CoV-2pp, CCoV-HuPn-2018pp, HCoV-229Epp, or VSVpp at 37°C for 3 h. Then, the viruses and neuraminidases were removed and replaced with fresh medium for further incubation. Mock-treated cells were incubated with DMEM and underwent the same manipulations as NA-treated cells. At 24 hpi, luciferase activity was measured, and relative infection is the ratio of the luciferase activity in cells treated with neuraminidases over that in the mock-treated cells.

For endosomal inhibitor assays, Huh7 cells or feline APN-transfected 293T cells in 96-well plates were infected with pseudotyped viral particles of VSVpp, CCoV-HuPn-2018pp, or HCoV-229Epp in the absence (mock) or presence of the indicated concentrations of NH_4_Cl, E-64d (Sigma-Aldrich, Cat. No. E8640), cathepsin L inhibitor (Sigma-Aldrich, Cat. No. 219427), cathepsin B inhibitor CA-074 Me-Calbiochem (Sigma-Aldrich, Cat. No. 205531), and cathepsin S inhibitor Z-FL-COCHO (MCE, Cat. No. HY-15533). Cells were lysed, and luciferase activity was measured at 24 hpi. Pseudotype entry is the ratio of the luciferase activity of inhibitor-treated cells over mock-treated cells.

### Virus-cell attachment assay

For the pseudotyped viral particles binding and attachment assay, Huh7 cells or feline APN and human ACE2 cotransfected 293T cells were pretreated with or without neuraminidase for 2 h at 37°C, and then the cells were inoculated with HIV-based pseudotyped lentiviral particles of IAVpp, SARS-CoV-2pp, CCoV-HuPn-2018pp, HCoV-229Epp, or VSVpp 2 for 1 h at 4°C. After extensive washing to remove unbound viruses, the cells were immediately harvested. The amounts of cell-associated viral RNA were quantified by qRT-PCR (Abbott RealTime HIV-1 Qualitative Assay, Cat. No. 4N66-90).

### Trypsin treatment

Huh7 cells or feline APN-transfected 293T cells were seeded into 96-well plates at 37°C overnight and then infected with pseudotyped viral particles of VSVpp, CCoV-HuPn-2018ppp, and HCoV-229Epp by centrifuging at 4°C at 3,500 × *g* for 35 min. Infected cells were treated with the indicated concentrations of TPCK-trypsin (Sigma-Aldrich, T1426; 0, 1, 2.5, 5, 7.5, 10 µg/mL) or DMEM without FBS at 37°C for 13 min. Then, DMEM, including 10% FBS, was added to the culture at 37°C for 24 h. Luciferase activities were measured at 24 hpi. Pseudotype entry is normalized to the infection efficiency of pseudotyped particles without viral envelope protein.

### Effect of TMPRSS2 on cellular entry

Huh7 cells in 96-well plates were transfected with the pCAGGS-TMPRSS2 plasmid or an empty pCAGGS plasmid as a negative control by Lipofectamine 2000 (Invitrogen). 293T cells in 6-well plates were cotransfected with 3 µg feline APN plasmid and 1 µg pCAGGS-TMPRSS2 plasmid or 1 µg empty pCAGGS plasmid as a negative control. At 24 hr post-transfection, 293T/fAPN and 293T/fAPN-TMPRSS2 cells were seeded into 96-well plates. One day later, the cells were infected with pseudotyped viruses. At 24 hpi, luciferase activities were measured by a luminometer.

### Phylogenetic analysis

Multiple alignments of amino acid sequences of the S gene of coronaviruses or nucleotide sequences of APN orthologs were performed using Clustal X (55). Phylogenetic trees were constructed using the neighbor-joining algorithm implemented in MEGA7.0.26 software. The tree is drawn to scale with branch lengths that reflect the evolutionary distances among the tested sequences. The evaluation of statistical confidence in nodes was based on 1,000 bootstrap replicates. Branches with <50% bootstrap value were collapsed. Reference sequences were obtained from the National Center for Biotechnology Information databases, and reference numbers are indicated as follows: CCoV-IIb 7/2020/AUS (QQY97020.1); CCoV-II 341/05 (ACJ63231.1); CCoV-II 174/06 (ACJ63242.1); HuCCoV_Z19 (QWY12682.1); CCoV-HuPn-2018 (QVL91811.1); TGEV-Purdue (DQ811789.2); PRCV/USA/Minnesota-46140/2016 (ASV45148.1); FCoV-ZJU1617 (QSL97039.1); feline infectious peritonitis virus (FIPV) 79–1146 (DQ010921.1); CCoV-IIa NA/09 (AEE69322.1); CCoV-II CB/05 (AAZ91437.1); FCoV-II WSU 79–1683 (JN634064.1); CCoV-IIa TN-449 (JQ404410.1); CCoV-IIa HLJ-073 (KY063618.2); CCoV-IIc A76 (JN856008.2); ferret coronavirus (FRCoV)-NL-2010 (AKG92640.1); ferret enteric coronavirus (FECV) MSU-2 (ADD49358.1); ferret systemic coronavirus (FRSCV) MSU-1 (ADD49350.1); FECV1 (ASR18938.1); FCoV-ZJU

Sequence alignment was performed using the ClustalW method to determine amino acid or nucleotide identities between the sequences of Tb bat and other species of bats. Reference sequences were obtained from the National Center for Biotechnology Information databases, and reference numbers are indicated as follows: *Artibeus jamaicensis* (Jamaican fruit-eating bat): XP_037000911.2; *Desmodus rotundus* (common vampire bat): XP_024417425.1; *Eptesicus fuscus* (big brown bat): XP_027994437.1; *Hipposideros armiger* (great roundleaf bat): XP_019495554.1; *Miniopterus natalensis*: XP_016072651.1; *Molossus molossus* (Pallas’s mastiff bat): XP_036133941.1; *Myotis brandtii* (Brandt’s bat): XP_005885265.1; *Myotis davidii*: XP_015418719.1; *Myotis lucifugus* (little brown bat): XP_014304588.1; *Myotis myotis*: XP_036211683.1; *Phyllostomus discolor* (pale spear-nosed bat): XP_028358239.1; *Phyllostomus hastatus* (greater spear-nosed bat): XP_045680750.1; *Pipistrellus kuhlii* (Kuhl’s pipistrelle): XP_036271433.1; *Pteropus alecto* (black flying fox): XP_006905412.1; *Pteropus giganteus* (Indian flying fox): XP_039714358.1; *Pteropus vampyrus* (large flying fox): XP_011377952.1; *Rhinolophus ferrumequinum* (greater horseshoe bat): XP_032956109.1; *Rousettus aegyptiacus* (Egyptian rousette): XP_016007055.1; and *Sturnira hondurensis*: XP_036909514.1.

### Homology-based structural modeling

Canine APN (PDB accession no. 7U0L) in the bound conformation was extracted from the CCoV-HuPn-2018 RBD/canine APN complex and used as a template for homology modeling (29). APN homology models were generated using the one-to-one threading algorithm of Phyre2 (56). The models were then aligned and compared to the intact CCoV-HuPn-2018 RBD/APN complex in PyMOL (The PyMOL Molecular Graphics System, Version 2.0; Schrödinger, LLC).

### Statistical analysis

All the experiments were repeated at least three times. Differences between the control sample and tests were statistically analyzed using unpaired two-tailed Student’s *t*-tests or one-way analysis of variance (ANOVA) by GraphPad Prism (v.8). *P* values less than 0.05 were considered statistically significant. *, *P* < 0.05; **, *P* < 0.001.

## SUPPLEMENTAL MATERIAL

Fig. S1 to S4 jvi.00601-23-s0001.docx Supplemental figures and legends.

ASM does not own the copyrights to Supplemental Material that may be linked to, or accessed through, an article. The authors have granted ASM a non-exclusive, world-wide license to publish the Supplemental Material files. Please contact the corresponding author directly for reuse.
