# Supplementary material for: Characterization of CCoV-HuPn-2018 spike protein-mediated viral entry
Source: J Virol. 2023 Sep 28;97(9):e00601-23. doi: 10.1128/jvi.00601-23 (PMC10537617; doi:10.1128/jvi.00601-23)
Supplement: Fig. S1 to S4 — Supplemental figures and legends. [file jvi.00601-23-s0001.docx]

**Supplemental Figures (Figures S1 to S4)**


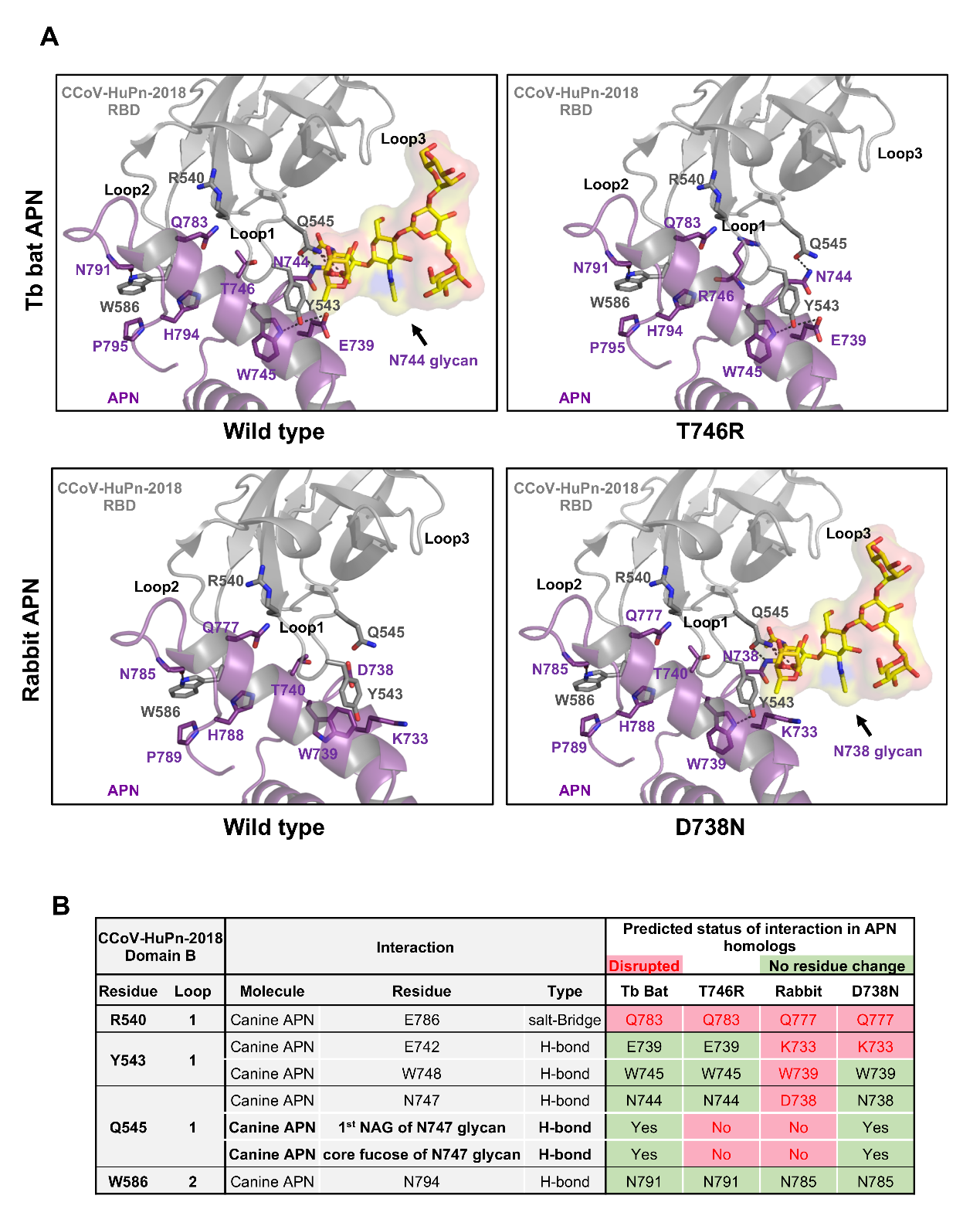


**Fig. S1. Critical RBD-binding residues in APN orthologs.** (A) Structural models of key residues at the contact interface between Tb bat APN or rabbit APN, and the RBD of CCoV-HuPn-2018. Canine APN (PDB accession no. 7U0L) in the bound conformation was extracted from the CCoV-HuPn-2018 RBD/canine APN complex and used as a template for homology modeling (1). APN homology models were generated using the one-to-one threading algorithm of Phyre2 (2). The models were then aligned and compared to that of the intact CCoV-HuPn-2018 RBD/APN complex in PyMOL. The electron density around the N744 glycan of Tb bat APN and N738 glycan of Rabbit APN are shown as a mesh of red and yellow colors. (B) Predicted effect of critical residue substitutions in APN orthologs on the interaction with the RBD of CCoV-HuPn-2018. Disruptive residues are colored in pink, and green indicates no residue change. The effects of residue substitutions were predicted by homology-based modeling analyses based on the crystal structure of the CCoV-HuPn-2018 RBD/ canine APN complex (1).


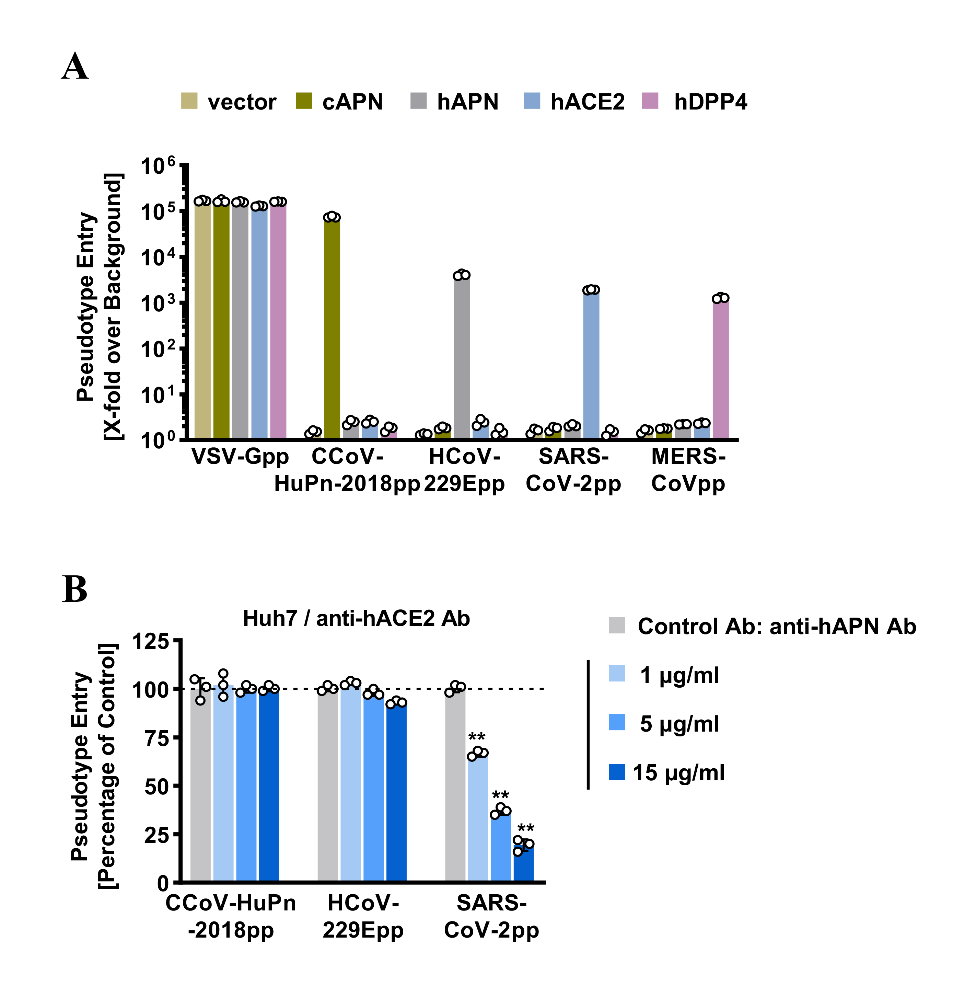


**Fig. S2. hACE2 and hDPP4 are not entry receptors for CCoV-HuPn-2018.** (A) Pseudotyped virus entry. 293T cells transfected with plasmid DNA of empty vector, canine APN (cAPN), human APN (hAPN), human ACE2 (hACE2), or human DPP4 (hDPP4) were infected with the indicated pseudoviruses. At 24 h post-infection, the luciferase activities were measured, and pseudotype entry was normalized against pseudotyped particles without viral envelope protein. (B) Entry inhibition assay with anti-hACE2 antibody. Huh7 cells were pre-incubated with indicated concentration of anti-hACE2 antibody (hAPN antibody served as a control antibody) for 1 h, and then infected with the indicated pseudoviruses in the presence of indicated concentration of hACE2 antibody or hAPN antibody for another 3 h, and then the virus and antibodies were removed and replaced with fresh medium for further incubation. At 24 h post-infection, luciferase activity was measured, and pseudotype entry was normalized against the control antibody-treated cells. Error bars reveal the standard deviation of the means from three biological repeats. **, *P* < 0.001 compared to the level of control antibody.


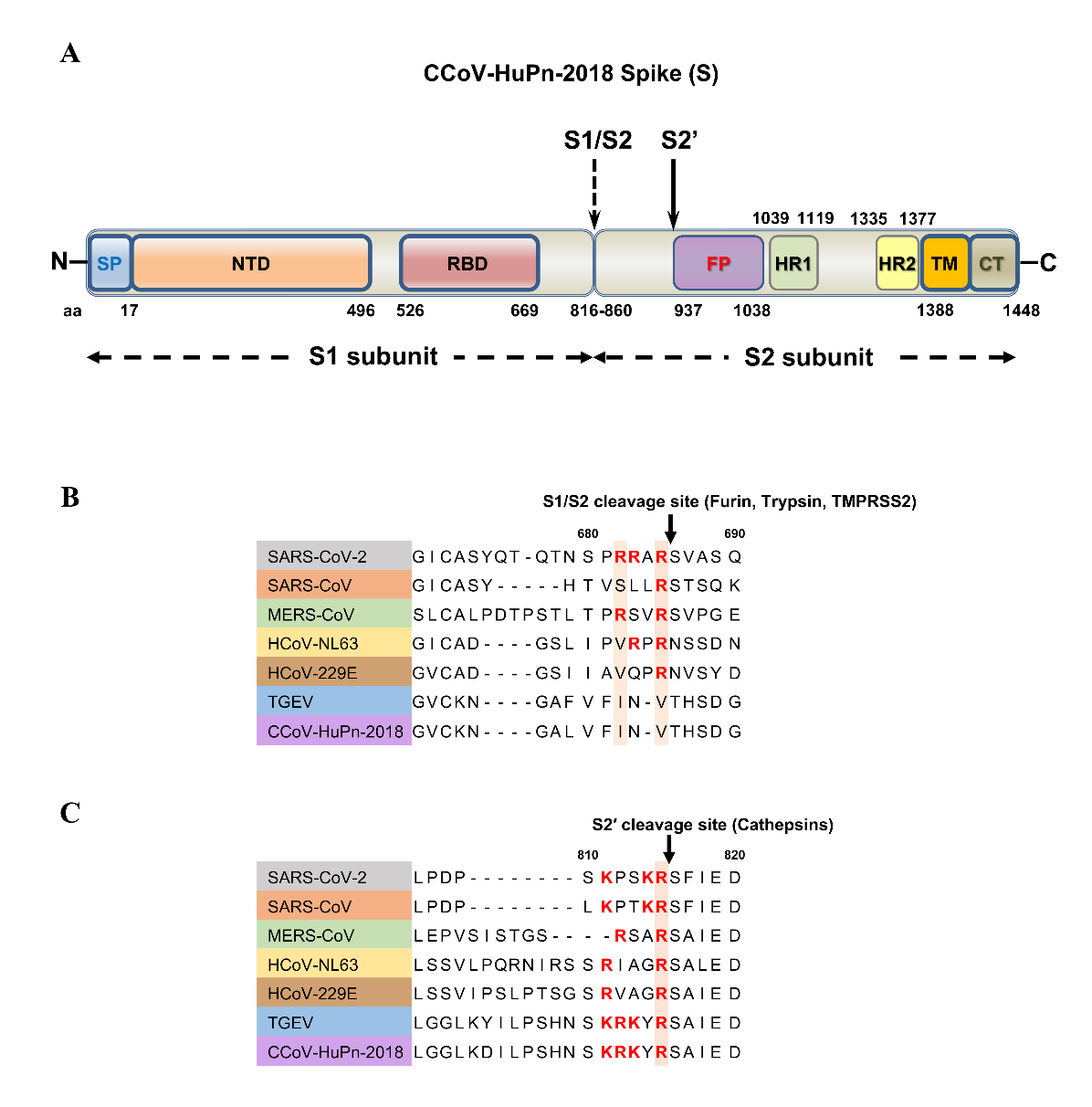


**Fig. S3. Protease cleavage sites in in the S protein of CCoV-HuPn-2018.** (A) Schematic diagram of the CCoV-HuPn-2018 spike (S) protein organization. SP, signal peptide; NTD, N- terminal domain; RBD, receptor-binding domain; FP, fusion peptide; HR1, heptad-repeat 1; HR2, heptad-repeat 2; TM, transmembrane domain; CT, cytoplasmic tail. Dashed arrow indicates the putative furin/trypsin/serine protease cleavage site at S1/S2 boundary, and solid arrow indicates cathepsin cleavage site at S2’. (B and C) Sequence alignment of several coronavirus S sequences at the proposed S1/S2 (B) and S2’ (C) position. Sequence alignment was performed with Clustal W. Amino acids contributing to monobasic or polybasic cleavages sites are shaded or colored in red.


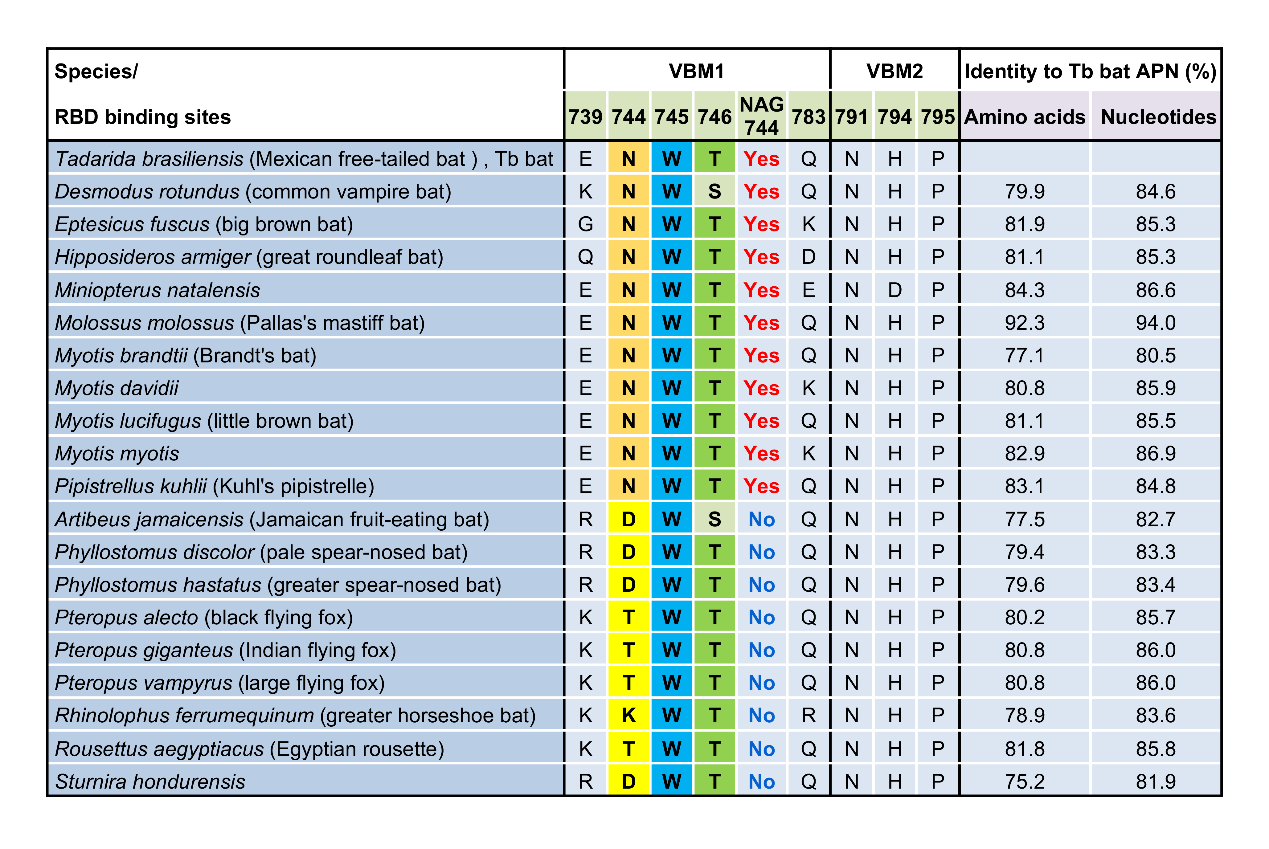


**Fig. S4. Critical RBD-binding residues in bat APNs.** Amino acid variations at seven potential CCoV-HuPn-2018 RBD-binding sites in the sequences of twenty bat APNs. The columns on the left are amino acid sequence alignment profiles of twenty species of bat APNs, focusing on the seven potential RBD-binding sites and N744 glycan (Tb bat APN numbering). The columns on the right are identities between Tb bat APN and other species of bat APNs for their amino acid sequences and nucleotide sequences. One-letter codes are used for amino acids, and amino acids of the “N-W-T/S” motif are highlighted in different colors. VBM, virus binding motif.

**Reference**

1. Tortorici MA, Walls AC, Joshi A, Park YJ, Eguia RT, Miranda MC, Kepl E, Dosey A, Stevens-Ayers T, Boeckh MJ, Telenti A, Lanzavecchia A, King NP, Corti D, Bloom JD, Veesler D. 2022. Structure, receptor recognition, and antigenicity of the human coronavirus CCoV-HuPn-2018 spike glycoprotein. Cell 185:2279-2291 e17.

2. Kelley LA, Mezulis S, Yates CM, Wass MN, Sternberg MJ. 2015. The Phyre2 web portal for protein modeling, prediction and analysis. Nat Protoc 10:845-58.
